# Antibiotic Resistance in Pediatric Infections: Global Emerging Threats, Predicting the Near Future

**DOI:** 10.3390/antibiotics10040393

**Published:** 2021-04-06

**Authors:** Alessandra Romandini, Arianna Pani, Paolo Andrea Schenardi, Giulia Angela Carla Pattarino, Costantino De Giacomo, Francesco Scaglione

**Affiliations:** 1 Department of Oncology and Hemato-Oncology, Postgraduate School of Clinical Pharmacology and Toxicology, Università degli Studi di Milano, 20122 Milan, Italy; alessandra.romandini@unimi.it (A.R.); paolo.schenardi@unimi.it (P.A.S.); 2 Department of Oncology and Hemato-Oncology, Università degli Studi di Milano, 20122 Milan, Italy; arianna.pani@unimi.it (A.P.); francesco.scaglione@unimi.it (F.S.); 3 Maternal and Infantile Department of Pediatrics, ASST Grande Ospedale Metropolitano Niguarda, 20122 Milan, Italy; giuliaangelacarla.pattarino@ospedaleniguarda.it

**Keywords:** multidrug resistance, antibiotics, antibiotic resistance, childhood, infections, antimicrobial stewardship

## Abstract

Antibiotic resistance is a public health threat of the utmost importance, especially when it comes to children: according to WHO data, infections caused by multidrug resistant bacteria produce 700,000 deaths across all ages, of which around 200,000 are newborns. This surging issue has multipronged roots that are specific to the pediatric age. For instance, the problematic overuse and misuse of antibiotics (for wrong diagnoses and indications, or at wrong dosage) is also fueled by the lack of pediatric-specific data and trials. The ever-evolving nature of this age group also poses another issue: the partly age-dependent changes of a developing system of cytochromes determine a rather diverse population in terms of biochemical characteristics and pharmacokinetics profiles, hard to easily codify in an age- or weight-dependent dosage. The pediatric population is also penalized by the contraindications of tetracyclines and fluoroquinolones, and by congenital malformations which often require repeated hospitalizations and pharmacological and surgical treatments from a very young age. Emerging threats for the pediatric age are *MRSA*, *VRSA*, ESBL-producing *Enterobacteriaceae*, carbapenem-resistant *Enterobacteriaceae* and the alarming colistin resistance. Urgent actions need to be taken in order to step back from a now likely post-antibiotic era, where simple infections might cause infant death once again.

## 1. Introduction

At present times, antimicrobial resistance (AMR) is one of the most important public health threats worldwide and should be considered a high priority by all healthcare workers and institutions.

To understand the relevance of the threat posed by AMR, WHO estimated that every year in the world, infections caused by multidrug resistant (MDR) bacteria result in 700,000 deaths across all ages, of which around 200,000 are newborns [1]. In Europe, MDR infections in pediatric patients may represent up to 30% of the total cases [2]. In regions of the Middle East, 90% of newborns with sepsis, hospitalized in ICU, had resistant bacteria [3]; in some areas of South East Asia, 83% of children have *E. coli* resistant to first line antibiotics [4]; in Sub-Saharan Africa, 66% of neonatal sepsis and meningitis were found to be caused by bacteria resistant to antibiotics [5]; in a USA study, 20% of pediatric patients receiving colistin to treat already MDR Gram negative bacteria developed resistance [6].

The prevalence of multidrug-resistant organisms (MDROs) is surging up and is associated with a significant morbidity and mortality in affected patients. Infections caused by MDR bacteria are more difficult to treat and are related to a more severe and prolonged illness that leads to longer hospitalization times, with a 20% increase of length of stay and a poorer outcome, raising mortality by up to 40% in MDR hospital-acquired infection [7,8,9,10]. This translates also into a monetary cost with a deep impact on healthcare systems, with 2.39 billion dollars estimated to have been spent to treat MDR infections in the USA alone [11,12].

This problematic condition for our present poses a threat to our future and is linked with causes that are diverse and interconnected. Even though Alexander Fleming had warned the scientific and medical community regarding antibiotic overuse in 1945, still, an era of antibiotic abuse in agriculture, livestock, veterinary, and human medical practices started after World War II. This abuse was the leading driver of the evolution of resistance in bacteria [13,14]. Furthermore, medical malpractice contributed to select MDR strains of bacteria through unnecessary, inappropriate or suboptimal prescribing, which has been observed in 30% to 60% of the antibiotic therapies given to outpatients as well as to inpatients in some studies [15,16].

These behaviors are accelerating an already quick natural selection of MDROs. If we consider the time elapsed between the introduction of a new antibiotic in the market and the appearance of MDROs for that drug, we can observe how the antibiotic with the longest period of time without resistance was vancomycin with 16 years. But this interval drops to only two years for penicillin, and even one year for the more recent daptomycin and ceftaroline [17,18].

In such a quickly evolving context, without urgent actions we are headed towards a post-antibiotic era where common infections might once again be fatal, and this is especially true for children, who are frequently exposed to common infections, and consequently to a selective antimicrobial pressure after the perinatal period. This is particularly true also for newborns younger than a week, because of the presence of MDR bacteria populations in their gastrointestinal tract probably due to the exposure to environmental and maternal bacteria during and immediately after delivery [19,20]. According to a projection made by the WHO, if this trend is not reversed, drug-resistant diseases could cause 10 million deaths each year by 2050, with an estimated annual cost for healthcare systems and gross domestic product loss of $300 billion [1,7]. It is a burden on our society that must be limited by promoting antimicrobial stewardship and halting antibiotic abuse in any field.

For these compelling reasons, we conducted a review on the current pediatric AMR, its emerging threats, and potential strategies to overcome it.

## 2. The Roots of Antibiotic Resistance in Childhood

Children are known to receive antibiotics more often than any other type of drug, as they are frequent subjects of infections of various etiologies, from the more common urinary tract infections to the less frequent meningitis.

### 2.1. Antibiotic Overuse and Misuse in Hospitalized Children and Outpatient Care

The improper use of antibiotics is the most important cause of the modern expansion of antibiotic resistance [21,22]. The roots of improper antibiotic use are the poor knowledge of pathogens associated with different infections, and of pharmacokinetic and pharmacodynamic characteristics of the specific antibiotic classes. These features highly influence and affect drug choice, correct doses, posology and duration of a treatment course. In addition, antibiotics are still commonly prescribed for wrong diagnoses, such as viral infections, especially in outpatient care [23,24,25].

In 2012, the Worldwide Antibiotic Resistance and Prescribing in European Children (ARPEC) group obtained detailed information on antibiotic use in hospitalized neonates and children from a multicenter retrospective cohort study for the first time. More than 17,000 pediatric hospital admissions in 226 hospitals from 41 countries all over the world, of which 6499 inpatients received at least one antimicrobial, were analyzed. They showed high level of use of broad-spectrum antibiotics in certain regions, possibly explained by high incidence of ESBL-producing or carbapenem-resistant Gram-negative organisms. However, the high level of empirical broad-spectrum antibiotic use may indicate that at least a portion of this prescribing may be inappropriate. Such inappropriate use was also found by Levy et al. [26] in pediatric intensive care units (PICUs) and pediatric wards, which the authors attributed to failure to discontinue or de-escalate therapy. In poorer and resource-limited settings, where de-escalation can be less considered and where bacteriological cultures are less often performed, successful de-escalation of carbapenems has been reported nonetheless [27]. The ARPEC study also found a significantly high use of amikacin in neonates admitted to Western European, Southern European, Asian and Latin American hospitals, and meropenem was prescribed at an alarmingly wide scale to Asian newborns.

On the other hand, a striking regional difference in antibiotic prescribing was observed among hospitalized children (older than 28 days). A high proportion of African, Australian, Western European and Northern European children continued to receive older narrow-spectrum antibiotics, mainly benzylpenicillin, sulfamethoxazole/trimethoprim, amoxicillin and gentamicin. In Eastern and Southern Europe, Asia, North and Latin America, children received more broad-spectrum antibiotics, mainly third-generation cephalosporins and carbapenems. Thus, it seems that resource-limited settings favor the empiric employment of older narrow-spectrum antibiotics, while developed countries (thus, hospitals) prefer to prescribe new-generation antibiotics, which offer a broader spectrum.

In conclusion, the 2016 ARPEC study identified potential quality indicators of correct antimicrobial use in pediatric populations:

- prevalence of broad-spectrum agents use
- discontinuation or de-escalation of antibiotics according to microbial culture results
- rates of antibiotic use by ward, hospital or nation
- early switch from parenteral to oral therapy
- good documentation of the reason for prescription in the patient’s medical charts
- <24 h perioperative administration of antibiotics for surgical prophylaxis.

### 2.2. Posology and Dose Appropriateness for the Pediatric Age

The pediatric population deserves pharmacological treatments that meet their needs, in terms of appropriate prescription, safety, posology and efficacy profiles. This is especially true when the great pediatric variability of pharmacokinetics (PK), which is in part age-dependent, is taken into consideration.

The lack of specific pediatric clinical trials on antibiotics and the common practice of complete or partial extrapolation of adult pharmacokinetic and pharmacodynamics (PD) data to the pediatric population (with weight or body surface area as the only discerning indicator) contribute to a scarcity of high-evidence-level knowledge and strictly pediatric guidelines. Even though extrapolation of drugs’ efficacy data to the pediatric population is an important tool to reduce the need for pediatric efficacy trials, dose-finding and safety pediatric trials are still crucial to offer the best standard of care to this important population. The practice of extrapolation and the lack of specific pediatric clinical trials on antimicrobials can often cause antibiotic misuse, over- or under-dosage, leading to a relative risk of toxicity and/or to a development of antibiotic-resistant bacterial strains specific to the pediatric age.

In particular, the complex variability of PK comes from the great range of pediatric age groups, from premature to neonates and from infants and children to adolescents. While age-related physiologic development continues into childhood and adolescence, the most prominent differences, compared with adults, are in neonates and infants. This is true especially considering kidney function, which has been proven to be extremely variable and age-dependent: the glomerular filtration rate increases quickly through the first two weeks of life and then rises steadily until adult rates are reached at 8–12 months of age [28]. Moreover, tubular secretion is immature at birth and reaches adult function throughout the first year of life. This kidney function developmental variability directly correlates with a highly diverse drug clearance through the pediatric age.

Strategies to optimize pediatric dosing, preventing ethical issues and specific pediatric difficulties in conducting trials on this population [29], have been put forward. In addition, there are practical difficulties which should be taken into consideration when approaching such a specific age group, such as child-friendly formulations and feeding regimens.

Developmental pharmacology has indeed advanced in recent decades and has provided more specific indications on the effects of maturation on absorption, elimination and effects of antibiotics. Nevertheless, further studies are needed to better understand PK and PD in the pediatric population so that antibiotic prescription can live up to the best standards of care, especially in children with underlying conditions. In particular, the population pharmacokinetic–pharmacodynamics method is ideal for this population, as it requires only a few samples per patient at flexible times [29]. It describes both the average behavior of a homogeneous population and the interindividual differences. The use of the population PK–PD method on this population has been widely endorsed by regulatory agencies and is highly detailed in the Guidance Documents of the Food and Drug Administration (FDA) and European Medicines Agency (EMA) [30,31]. Another proposed approach to characterize drugs’ uptake, effects and PK in this population is the physiologically based pharmacokinetic (PBPK) model, a promising method to support first-time dosing studies in children [32]. This model combines the developmental physiological processes of children with adult PK data: they require adult PK parameters and pediatric-specific information on the anatomical, physiological and biochemical variables from birth to age 18. The use of PBPK models has been advocated to support first-time dosing in children as well as in the design of pediatric clinical trials [33,34].

Despite the recent developmental pharmacology advances, there still is a strong need for more research on the development of drug metabolizing enzymes, transporters, receptor system and disease course. As knowledge on pediatric developmental physiology grows, drugs will be prescribed to children with greater accuracy, efficacy and safety.

### 2.3. Contraindicated in Children: Sometimes, It Is a Matter of a Lack of Options

It is also relevant to note that some antibiotics have precise contraindications when it comes to pediatric age, and this affects the range of antimicrobial options to treat infections.

For instance, fluoroquinolones remain largely not used in children due to safety concerns. These antibiotics have shown irreversible adverse events on cartilage development in young animals through the inflammation and disruption of weight-bearing joints [35,36]. The potential occurrence of these severe arthropathies has limited the use of fluoroquinolones in children, as these events have been clearly reported in various pediatric studies comparing fluoroquinolones to non-fluoroquinolone treatments. One additional concern about fluoroquinolone use is their predisposition to create increased bacterial resistance. In the past 25 years, resistance patterns to fluoroquinolones have significantly worsened in adults, and may become an important concern in the pediatric population with persistent use. Fluoroquinolones have nonetheless been used successfully in a variety of pediatric infections, including cystic fibrosis exacerbations, though most of the literature supporting the use of fluoroquinolones in children comes from retrospective or small, uncontrolled studies. Benefits and risks of this antibiotic therapy must be carefully weighed. In 2006, the American Academy of Pediatrics (AAP) recommended fluoroquinolones only in three major circumstances: (1) FDA-approved indications; (2) MDROs with no safe or effective alternative; and (3) oral fluoroquinolone sensitivity when all other options are intravenous only. They may be considered in pediatric patients with gastrointestinal infections, acute otitis media, sinusitis, lower respiratory tract infections, pneumonia or *Mycobacterium* infections [37].

Another class of antibiotics largely unused in the pediatric age is represented by tetracyclines. Tetracyclines were first introduced in 1948 with some warnings for tooth discoloration in children already reported in 1956 [38]. This event is more likely to occur during the tooth-calcification process, completed by age eight [39], thus this class of antibiotics is relatively contraindicated in children aged less than eight years. In older children, tetracyclines have been successfully used for respiratory infection, community-acquired *S. aureus* resistant to methicillin (*MRSA*), malaria and acne. Of note, another adverse event is tetracycline-induced photosensitivity, which usually manifests as a photosensitive rash, similar to an intense sunburn. Phototoxicity can represent a significant concern, though the permanent tooth discoloration is a sufficient reason to avoid the prescription of these antibiotics in young children whenever possible. When tetracycline use is essential, limited evidence suggests that minimizing the total dose and length of exposure and employing the lower calcium-binding tetracycline (e.g., doxycycline) may reduce the risk of tooth discoloration. The AAP recommended the use of tetracycline in pediatric infections when the benefits outweigh the risks of adverse events. Their indications include rickettsial infections, cholera, anthrax and they suggest prescribing doxycycline, as it has a lower risk of dental staining with less frequent posology intervals [40].

### 2.4. Two Modern Infection-Predisposing Issues: Biofilms and Chronic Conditions

Biofilms are associated with chronic subsequent infections and with an inherent resistance to antibiotic therapy, due to the bacteria adhering to the damaged tissue or implanted medical device. Bacteria in biofilms persist by a strategy of tenacious survival rather than aggressive virulence. Biofilm infections can linger for months or even years, rarely being fatal but often sitting undisturbed by antibiotic treatment.

Treatment of infections caused by colonization of biofilms often fails as these infections require high antibiotic doses for a prolonged time. The known mechanisms of antimicrobial resistance, such as modifying enzymes, target mutations and efflux pumps, do not seem to be associated with the protection of bacteria in biofilms. In fact, when bacteria are dispersed from biofilm, they usually become sensitive to antibiotics, which suggests an inherent antibiotic resistance due to the peculiar habitat this film of cells creates. Conventional antibiotic resistance mechanisms fail to clarify most cases of antibiotic-resistant biofilm infections, so three main hypotheses may explain this phenomenon. The first hypothesis is the chance of slow or incomplete penetration of the antibiotic into the biofilm [41]; the second hypothesis depends on an altered chemical microenvironment within the biofilm [42]; a third mechanism of antibiotic resistance is a subpopulation of bacteria in a biofilm shifting to a unique, highly protected phenotypic state—differentiating into “persister cells”. Persister cells are a subgroup of temporarily antibiotic-tolerant bacterial cells, often with a slow or arrested growth, and are able to continue growth after severe stress. They are associated with recurrent persistent bacterial infections and are linked to an increased risk of AMR [43].

It has been found that children can be exceptional hosts of biofilms. They are the recipients of medical devices that can be as simple as central venous catheters up to complex heart defect patches and ventriculoperitoneal shunts. These devices can be a formidable breeding ground for biofilms and bacteria, virtually unreachable by antibiotics. These may predispose to long-term infections, which may sometimes be definitively treated only through the removal and replacement of the device.

Broadly speaking, a relevant portion of infections in children are correlated to biofilms, even in the absence of a biomedical device. Biofilms have also been found in the nasopharynx of children affected with repeated upper airways and middle ear infections, which may explain the frequent lack of efficacy of antibiotic therapy in these cases [44]. In particular, biofilms have been recognized as responsible for recurrent acute otitis media and otitis media with effusion, a finding that may be explained by the evidence that otitis media with effusion frequently presents with a negative culture despite a positive molecular research for pathogens, is often unresponsive to antibiotics and is highly recurrent. Moreover, children affected with chronic rhinosinusitis are more prone to have biofilms than children with no underlying condition or children with acute rhinosinusitis, so much so that mature biofilms were found in 95% of adenoids removed from children affected with chronic rhinosinusitis [45].

Chronic conditions, such as cystic fibrosis (CF), are associated with significant morbidity and early mortality due to recurrent acute and chronic infections that may either occur because of the precise pathophysiology of the disease, and/or of frequent and prolonged hospitalizations, as the chronic use of multiple antibiotics increases the chance of MDR. In particular, pediatric CF patients seem to harbor different opportunistic infections compared to adults and this is due to how different bacteria use type VI secretion systems to mediate interbacterial competition [46]. This also impacts antibiotic treatment in young and adult CF patients, and may explain the emergence of AMR.

## 3. Emerging Threats

The WHO recently published a list of bacteria for which new antimicrobials are urgently needed [47]. This comes from the observation of a progressive depauperation of the R&D pipeline of new antibiotics, in dire contrast with the concerning rise of resistant infections. The most urgent needs in terms of antibiotics R&D for the pediatric population are oral formulations for community-acquired infections with high morbidity, like drug resistant *ESBL-producing Enterobacteriaeceae*, *Neisseria gonorrhoeae*, and *Salmonella typhi*.

### 3.1. Resistant Staphylococcus Aureus

Strains of *MRSA* were first identified as a threat in adult patients in the 1960s. *MRSA* infections were uncommon in children until the 1990s, when these resistant strains were noted in infections in adults and children who had no prior predisposing risk or healthcare institutions contact [48]. Thus, healthcare-acquired *MRSA* (HA-*MRSA*) began circulating together with community-acquired *MRSA* (CA-*MRSA*). [49]. Trimethoprim-sulfamethoxazole and clindamycin are often used in the treatment of CA-*MRSA*: resistance to trimethoprim/sulfamethoxazole is relatively uncommon, though clindamycin resistance has increased in the past decade [49]. To help eradicate *MRSA* carriage and limit its spread, the application of mupirocin ointment into anterior nares has been put in practice, often together with chlorhexidine baths: since these practices started, resistance to mupirocin and chlorhexidine have emerged [50].

Vancomycin has long been considered the last resort for treating *MRSA* infections, though in 1997 the first strain of vancomycin-intermediate susceptible *Staphylococcus aureus* (*VISA*) was isolated from a surgical wound of a Japanese child whose infection did not respond well to long-term vancomycin therapy [51]. Since then, vancomycin resistance has cast a dark shade upon anti-*MRSA* antibiotic treatment, especially for children, whose therapeutic options remain few, including daptomycin and linezolid. Nonetheless, daptomycin is of no use in treating lung infections as it binds surfactant with its consequent inactivation [52]. Interestingly, ceftaroline, which is often overlooked, has a good efficacy on *MRSA*, maintaining the safety of a cephalosporin with a variety of indications both on or off label [53], and has been approved for children older than two months of age without a recognized widespread resistance so far identified.

### 3.2. ESBL-Producing Enterobacteriaceae (ESBL-Ent) and Carbapenem-Resistant Enterobacteriaceae (CRE)

In the U.S.A., there has been a significant increase of *ESBL-Ent* infections in children, correlated with the spread of MDR ST131 CTX-M-producing *E. coli* strains [54]. The acquisition of *ESBL-Ent* has a great variability depending on country, age, healthcare exposure, organism and ESBL genotype. International studies carried out in hospital settings characterize an increase in *ESBL-Ent* colonization: younger gestational age, low birth weight, antibiotic use and prolonged mechanical ventilation are risk factors for this population [55]. Outside the neonatal age, risk factors for *ESBL-Ent* infections are comparable to those of adults, including antibiotic use, chronic clinical conditions, healthcare access and recurrent infections [56], while neurological diseases might be a predisposing risk typical of the pediatric population [57]. Therapeutic options for *ESBL-Ent* include piperacillin-tazobactam, ceftazidime-avibactam, cefepime, fluoroquinolones, aminoglycosides, tigecycline, fosfomycin and carbapenems, although these options remain limited for children by safety profiles and often unclear dosage guidelines. In addition, carbapenem-sparing strategies should be taken into consideration first to combat their overuse, and when possible, a step-down strategy should be employed after empirical therapy.

Decreased sensitivity to carbapenems might be due to either the production of carbapenemases, which are β-lactamases hydrolizing penicillins (cephalosporins, carbapenems and monobactams), or to the production of ESBL or to downregulated porins. Resistance to carbapenems in pediatric populations has been described as dramatically increasing in recent decades, and the 2013 CDC report addressed this threat as urgent, highlighting a 50% mortality rate in hospitalized patients with bloodstream *CRE* infection [16]. A significant increase in *CRE* frequency in some pediatric populations was noted, especially for the *Enterobacter* species, from 0% in 2000 to 4.5% in 2012, especially in ICU settings [58]. Therapeutic options for *CRE* are few, and pediatric options are further limited: the use of tigecycline is carefully weighed for people <18 years of age due to its safety profile; colistin and other polymyxins have optimal dosing issues for the pediatric population; oral fosfomycin may be used for *CRE* bladder infection, though dosing guidelines are available only for older children and adolescents [59]. Moreover, *ESBL-Ent* and carbepenemase-producing bacteria are often carriers of other plasmid-transmitted genes that confer resistance also to aminoglycosides, sulfonamides and fluoroquinolones, which characterizes these bacteria as true MDROs [60].

### 3.3. Colistin Resistance

In mid-1990s, the rise of MDR Gram-negative bacteria along with a plunge in new antimicrobials production active towards them pushed clinicians to reconsider old, disused antibiotics, such as fosfomycin and, more interestingly, colistin [61]. After being introduced, colistin was quickly replaced by newer, safer parenteral antibiotics in the 1970s due to reports of nephrotoxicity and neurotoxicity [62]. When colistin use decreased in the 1980s, resistance to it had hardly been documented and resistance mechanisms were still unclear [63], but as this drug was reintroduced in clinical pediatrics as salvage therapy for difficult-to-treat MDR Gram-negative infections, patterns of resistance were for the first time noticed and reported. While a chromosomically encoded resistance to colistin had already been reported, over the past decade intrinsic mutations or adaptive mechanisms, as well as the discovery of an mcr-1 gene conferring resistance via plasmid transfer, were better described [64].

In conclusion, colistin activity needs to be preserved, as it is one of the very last few drugs effective against carbapenem-resistant Gram-negative organisms. In order to do so, it should be employed in very limited cases in clinical practice, keeping in consideration its complicated PK and its narrow therapeutic window, especially in pediatric patients.

## 4. Future Perspectives: Stepping Back from the Edge

### 4.1. Overcoming

A first step needed for the control of AMR is the implementation of an antimicrobial stewardship strategy in acute care, long-term care and outpatient care settings. Antimicrobial stewardship is defined as “coordinated interventions designed to improve and measure the appropriate use of antibiotic agents by promoting the selection of the optimal drug regimen including dosing, duration of therapy and route of administration” [65]. Thus, actions that must be implemented in a pediatric context in order to avoid the emergence of AMR and reverse the actual trend should include:

- The creation of an institutionalized team of experts formed by infectious disease specialists, clinical pharmacologists, microbiologists and pediatricians able to draw up local guidelines, provide advice on antimicrobial use and also to implement educational interventions;
- Regular auditing and reviewing of local antimicrobial prescriptions;
- Pharmacodynamics/pharmacokinetic-guided dosing, considering physiological developmental processes of the child.

Regarding the last point, in pediatrics precision dosing is of extreme importance, especially for antimicrobials therapy. The use of a software that is able to predict the correct dosage taking into account the characteristics of the particular drug, the developmental changes in pharmacokinetic and pharmacodynamics based on the maturation stage and the actual clinical condition of the newborn, infant or child, should be implemented in clinical practice. In line with this, the EMA Guidelines on the role of pharmacokinetics in the development of medicinal products in the pediatric population recommends putting effort “into finding markers correlated to maturation-related changes in pharmacokinetics, making individualization of the dose possible between individuals, as well as within an individual over time” [66].

### 4.2. Rapid Diagnosis

Empirical treatments are the culprit for antibiotic misuse and overuse: 50% of antibiotic therapies are started inappropriately and without a correct identification of the etiologic agent [13]. A rapid diagnosis is essential for the correct management of infectious disease and quick and accurate antimicrobial susceptibility testing (AST) is essential to avoid the spread of AMR and to choose the right antimicrobial agent at the right time. To date, the evolution of technologies is favoring the implementation of new diagnostic tools for rapid AST. Methods and equipment applying nucleic acid amplification, integrating mass-spectrometry and biosensor-based AST, are being validated. Moreover, in most advanced diagnostic centers, third generation technologies of whole genome sequences (WGS) are available and are able to quickly identify pathogens and test for drugs’ susceptibility genes [67]. The ideal management of antimicrobials requires a rapid analysis able to identify the etiological agent and to quickly give answers regarding susceptibility testing. Furthermore, the determination of the best antimicrobial dose based on personalized pharmacokinetic parameters is essential to achieve the right concentrations, avoiding AMR emergence and spread. This is particularly true in a pediatric context, where intervention timing is essential and pharmacokinetic variability should be carefully considered.

### 4.3. New Antibiotics for Children

There are some new antibiotics approved for pediatric use in the clinical armamentarium directed against MDR pathogens, such as ceftaroline [68,69] and ceftazidime/avibactam [70]. Other novel antibiotics are showing interesting evidence of efficacy in the pediatric population, such as ceftolozane/tazobactam [71], tedizolid [72] or dalbavancin [73], but more clinical trials are needed to confirm such data.

In particular, besides safety and efficacy trials, specific pharmacokinetics studies should be implemented, in order to determine the best dose regimen among specific age populations instead of extrapolating it from adult dosages. This will allow us to administer the appropriate dosage, avoiding the onset of new resistances and thus preserving the few new weapons at our disposal. Furthermore, the whole scientific community should be committed to implementing pharmacokinetic studies in pediatric populations, even for old drugs that are reappearing in wards as a viable option against MDR pathogens. In addition, pharmaceutical companies should also be strongly encouraged to engage in the pharmacological development of new antibiotics by including children in trials.

## 5. Conclusions

Antibiotic resistance is a worldwide threat, raising the menace of a post-antibiotic era where children will once again die because of simple, previously treatable infections.

Specific pediatric issues, such as reckless antibiotic prescriptions for wrong diagnoses, the limited options, the lack of trials on children, and the evolving nature of this diverse population, are the culprits in this complex perspective.

A relatively new problem, subsequent to antibiotic introduction in the medical world, needs new solutions. These may include the creation of new clinical teams for antibiotic prescriptions in wards, the use of a software able to consider multiple variables and return a specific drug and dosage, the implementation of quick antimicrobial susceptibility tests to direct an empirical therapy, encouraging the use of new antibiotics and favoring the inclusion of children in both old and new drugs’ trials.

## Data Availability

Data sharing not applicable.
